# ^89^Zr-PET imaging of DNA double-strand breaks for the early monitoring of response following α- and β-particle radioimmunotherapy in a mouse model of pancreatic ductal adenocarcinoma

**DOI:** 10.7150/thno.44772

**Published:** 2020-04-27

**Authors:** Sophie Poty, Komal Mandleywala, Edward O'Neill, James C. Knight, Bart Cornelissen, Jason S. Lewis

**Affiliations:** 1 Department of Radiology, Memorial Sloan Kettering Cancer Center, NY, USA; 2 CRUK/MRC Oxford Institute of Radiation Oncology, Department of Oncology, University of Oxford, Oxford, UK; 3 School of Natural and Environmental Sciences, Newcastle University, Newcastle upon Tyne, UK; 4 Radiochemistry and Molecular Imaging Probes Core, Memorial Sloan Kettering Cancer Center, New York, NY, USA; 5 Weill Cornell Medical College, New York, NY, USA; 6 Molecular Pharmacology Program, Memorial Sloan Kettering Cancer Center, New York, NY, USA

**Keywords:** Radioimmunotherapy, DNA damage, γH2AX, PET, pancreatic cancer

## Abstract

**Rationale**: The evaluation of early treatment response is critical for patient prognosis and treatment planning. When the current methods rely on invasive protocols that evaluate the expression of DNA damage markers on patient biopsy samples, we aim to evaluate a non-invasive PET imaging approach to monitor the early expression of the phosphorylated histone γH2AX in the context of pancreatic cancer targeted radionuclide therapy. Pancreatic ductal adenocarcinoma has a poor patient prognosis due to the absence of curative treatment for patients with advanced disease. There is therefore a critical need for the fast clinical translation of new therapeutic options. In line with these observations, our group has been focusing on the development of radiotheranostic agents based on a fully human monoclonal antibody (5B1) with exceptional affinity for CA19.9, an antigen overexpressed in PDAC. Two on-going clinical trials resulted from these efforts, one with ^89^Zr (diagnosis) and one with ^177^Lu (β-particle therapy). More recently, we successfully developed and evaluated in PDAC mouse models a targeted α-therapy strategy with high clinical translation potential. We aim to expedite the clinical translation of the developed radioimmunotherapy approaches by investigating the early therapeutic response and effect of radiation therapy in a PDAC mouse model via PET imaging.

**Methods**: Mice bearing BxPC3 tumor xenografts were treated with α- and β-particle pretargeted radioimmunotherapy (PRIT), external beam radiotherapy (EBRT), or sham-treated (vehicle). The phosphorylated histone γH2AX produced as a response to DNA double strand breaks was quantified with the PET radiotracer, [^89^Zr]Zr-DFO-anti-γH2AX-TAT.

**Results**: PET imaging studies in BxPC3 PDAC mouse models demonstrated increased uptake of [^89^Zr]Zr-DFO-anti-γH2AX-TAT (6.29 ± 0.15 %IA/g) following β-PRIT in BxPC3 PDAC xenografts as compared to the saline control group (4.58 ± 0.76 %IA/g) and EBRT control group (5.93 ± 0.76 %IA/g). Similarly, significantly higher uptake of [^89^Zr]Zr-DFO-anti-γH2AX-TAT was observed in tumors of the ^225^Ac-PRIT and EBRT (10 Gy) cohorts (7.37 ± 1.23 and 6.80 ± 1.24 %IA/g, respectively) compared to the negative control cohort (5.08 ± 0.95 %IA/g). *Ex vivo* γH2AX immunohistochemistry and immunofluorescence analysis correlated with *in vivo* ^89^Zr-anti-γH2AX PET/CT imaging with increased γH2AX positive cell and γH2AX foci per cell in the treated cohorts. When α-PRIT resulted in prolonged overall survival of treated animals (107.5 days) as compared to β-PRIT (73.0 days), no evidence of difference in [^89^Zr]Zr-DFO-anti-γH2AX-TAT uptake at the tumor site was observed, highlighting that DNA damage is not the sole radiobiology paradigm and that off-targeted (bystander) effects should be considered.

**Conclusions**: PET imaging studies with [^89^Zr]Zr-DFO-anti-γH2AX-TAT following α- and β-particle PRIT in a BxPC3 PDAC subcutaneous xenograft mouse model allowed the monitoring of tumor radiobiological response to treatment.

## Introduction

With a 5-year survival rate in the US of 9%, pancreatic ductal adenocarcinoma (PDAC) is a uniformly lethal disease 1,2. This dire prognosis is partly due to a lack of screening and diagnostic tools, but also limited therapeutic options 1. To solve these issues, our group has been developing clinically translatable diagnostic and therapeutic approaches based on antibody radioconjugates for the positron emission tomography (PET) imaging and radioimmunotherapy (RIT) of PDAC 3-6.

The Sialyl-Lewis^a^ (sLe^a^), also known as CA19.9 antigen, is one of the most widely studied pancreatic cancer biomarkers as patients' serum levels correlate with cancer stage, prognosis or treatment response 7-9. The fully human 5B1 antibody, generated from blood lymphocytes from a patient immunized with a sLe^a^ - KLH vaccine, demonstrates exceptional affinity and high specificity for an extracellular epitope of CA19.9 10,11. In 2016, the clinical potential of CA19.9 as a therapeutic target was investigated in patients with pancreatic cancers or CA19.9-positive malignancies (NCT02672917) via the weekly intravenous administration of 5B1 (MVT-5873). This antibody was also evaluated as a platform for the specific delivery of radioisotopes to PDAC tumor sites. Two on-going clinical trials with 5B1-based radioimmunoconjugates resulted from these studies: one in which the antibody is labeled with ^89^Zr for PET imaging (NCT02687230) and a second in which the antibody is labeled with ^177^Lu for β-RIT (NCT03118349). More recently, our group evaluated the potential of ^225^Ac labeled 5B1-conjugates for α-RIT in PDAC mouse models 6. During these preclinical investigations, a pretargeted administration method relying on the inverse electron demand Diels-Alder reaction 4,5,12-15 was compared to conventional RIT. Pretargeted RIT (PRIT) allowed the delivery of α-radioactive payloads to PDAC tumor sites while reducing the mean absorbed dose to healthy tissues, enabling prolonged survival and reduced haematotoxicity in subcutaneous and orthotopic models of PDAC as compared to conventional RIT 6. The clinical translation of α-based PRIT for PDAC would complement the two on-going 5B1-based clinical trials for the diagnosis (^89^Zr) and β-therapy (^177^Lu) of PDAC and has the potential to extend the population of patients eligible for CA19.9-targeted radiotherapy to include those with residual metastatic lesions as well as patients that develop resistance to β-therapy.

To expedite the clinical translation of new targeted radiotherapies (for small molecule, peptide and antibody-based systems), we aim to validate a PET imaging platform for the evaluation of early treatment response. Conventional methods to assess therapeutic response rely on the monitoring of tumor dimensions through anatomic imaging techniques such as X-ray, CT or MRI 16,17. The visualization and quantification of cellular or biochemical responses to treatment through molecular imaging techniques offer the incredible opportunity for early treatment response monitoring when anatomical changes cannot be measured yet 18. The monitoring of therapeutic response after radiotherapy can be gauged by the quantification of DNA damage 19. DNA double-strand breaks (DSBs) are the most severe forms of DNA damage and one of the main mechanisms through which radiotherapy leads to apoptosis 20. As DNA DSBs are not present in large numbers, they do not represent an ideal target for molecular imaging. An alternative strategy lays in the targeting of DNA damage response proteins such as phosphorylated γH2AX which is present exclusively in the cell nucleus as foci containing thousands of copies around individual DNA DSBs 21-23. In a clinical setting, quantification of γH2AX foci is performed by the immunohistochemistry evaluation of biopsied samples 24. However, this method is invasive, associated with risks (e.g. bleeding, infection), not adequate for a disseminated disease and can be biased by tumor heterogeneity. At the preclinical stage, non-invasive SPECT (^111^In) and PET (^89^Zr) imaging techniques were developed to quantify γH2AX expression levels *in vivo*. Cornelissen et al. functionalized an anti-γH2AX antibody that binds both human and mouse γH2AX with a cell penetrating peptide (TAT) that contains a nuclear localization sequence and a chelator (DFO or DTPA) for further radiolabeling with ^89^Zr or ^111^In 25-27. The uptake of the SPECT imaging agent, [^111^In]In-anti-γH2AX-TAT, and the PET imaging agent, [^89^Zr]Zr-DFO-anti-γH2AX-TAT, was shown to be linearly dependent of the number of γH2AX foci per cell as well as the absorbed dose of radiation deposited at the tumor site by external beam radiation 25,27. The ability of [^89^Zr]Zr-DFO-anti-γH2AX-TAT to monitor early therapeutic response following chemotherapy was investigated in a PDAC mouse model 28. It was demonstrated that [^89^Zr]Zr-DFO-anti-γH2AX-TAT allows early indication of therapy response when the clinical standard, [^18^F]F-FDG, did not provide any indication after initiation nor completion of the treatment 28. Recently, [^111^In]In-anti-γH2AX-TAT allowed the non-invasive visualization of DNA damage following molecular targeted radionuclide therapy with [^177^Lu]Lu-DOTA-TATE in a preclinical mouse model of pancreatic neuroendocrine cancer 29. A dual isotope SPECT imaging strategy, allowing the simultaneous imaging of ^177^Lu and ^111^In, highlighted the correlation of the ^177^Lu dose heterogeneity to the uptake of [^111^In]In-anti-γH2AX-TAT. This study opened the path toward the evaluation of the DNA damage imaging approach for a larger range of targeted radionuclide therapy approaches including α- and β-RIT.

In this study, we investigated the ability of [^89^Zr]Zr-DFO-anti-γH2AX-TAT to monitor the activation of DNA damage response following α- and β-PRIT in a subcutaneous PDAC mouse model. We hypothesized that the *in vivo* quantification of γH2AX via PET imaging will provide an early readout of α-/β-PRIT efficacy. Such approach to early radiotherapy response is a critical tool for the clinical translation of new radiotherapy approaches, as it would ultimately expedite the translation evaluation time. Furthermore, this technology will be applicable to numerous radiotherapeutic delivery platforms, including small molecules, peptides, antibodies and nanoparticles, and will help streamline their development and translation.

## Materials and Methods

### Radiochemistry

The synthesis of DOTA-PEG_7_-Tz was performed using the previously published synthetic pathway 5. The conjugation of TCO-NHS to 5B1 was performed according to previously published methods 5,6. ^177^Lu was obtained from either ITG (Germany) or the University of Missouri Research Reactor through the United States Department of Energy Office of Science. ^225^Ac was supplied by the United States Department of Energy Office of Science by the Isotope Program in the Office of Nuclear Physics. ^177^Lu- and ^225^Ac-radiolabeling was performed according to protocols previously published by our group 5,30. ^225^Ac-radiopharmaceuticals for *in vitro* and *in vivo* evaluation were prepared and used at least 4 h after the purification of the radiotracers to allow actinium to reach a pseudo-equilibrium state and have an accurate reading of the activity used/injected. ^225^Ac samples from *in vitro* and *in vivo* assays were measured on a gamma counter once secular equilibrium was reached (> 24 hours).

The anti-γH2AX immunoconjugate for molecular imaging of DNA damage response was prepared following the published protocol 27. Succinctly, the free lysine residues of a mouse monoclonal anti-γH2AX antibody (Merck, clone JBW-301) were activated with an N-hydroxysuccinimidyl ester. The activated antibody was then conjugated to a TAT (GRKKRRQRRRPPQGYG) peptide, a cell penetrating peptide with a non-canonical nuclear localization sequence 25. The resulting immunoconjugate was then reacted with pSCN-Bn-deferoxamine (DFO) for further radiolabeling with ^89^Zr. ^89^Zr was produced through proton-beam bombardment of yttrium foil and isolated in high purity as ^89^Zr-oxalate at Memorial Sloan Kettering Cancer Center according to a previously published procedure 31. ^89^Zr-oxalate (10 MBq) was neutralized to pH 6.9-7.2 with 1 M Na_2_CO_3_. The DFO-anti-γH2AX-TAT in PBS buffer (pH 7.4) was added (100 μg), and the reaction was incubated at room temperature for 1 h with a gentle shaking (400 rpm).

Purity and radiolabeling efficacies were quantified through instant thin-later chromatography (iTLC) with a 50 mM ethylenediaminetetraacetic acid (pH 5, ^177^Lu and ^225^Ac), or a 0.1 M sodium citrate (pH 5.0, ^89^Zr) mobile phase. Radiochemical purity was routinely >99%.

### Cell lines and xenograft models

CA19.9-positive BxPC3 cells were grown in RPMI medium modified to contain 4.5 g/L sodium bicarbonate and supplemented with 10 % (vol/vol) heat-inactivated FCS, 100 IU penicillin, 100 μg/mL streptomycin, 10 mM HEPES, and 10 cc/L nonessential amino acids. All animals were treated according to the guidelines approved by the Research Animal Resource Center and Institutional Animal Care and Use Committee at Memorial Sloan Kettering Cancer Center. Female athymic homozygous nude mice (strain: Crl:NU(NCr)-Foxn^1nu^, Charles River Laboratories, Wilmington, MA, aged 6-8 weeks) were xenografted subcutaneously with 5×10^6^ BxPC3 cells suspended in 150 μL of a 1:1 Matrigel (Becton Dickson, Bedford, MA) and cell culture medium mixture.

### Cell viability

Cell viability 48 hours after [^177^Lu]Lu-DOTA-5B1 and [^225^Ac]Ac-DOTA-5B1 treatment was evaluated. BxPC3 cells (2x10^4^ cells) were seeded in 96 well plates and incubated for 4 h. [^177^Lu]Lu-DOTA-5B1 (220 MBq/mL to 0.55 kBq/mL) and [^225^Ac]Ac-DOTA-5B1 (2.97 MBq/mL to 7.4 Bq/mL) were added to the cells for 48h. Each concentration was run as quadruplicates. After 48 hours of treatment, a tetrazolium salt MTT (10 μL, 50 μg/mL) was added and incubated at 37°C for 1 hour. After that, a 0.04 M HCl solution in 10% Triton-x isopropanol (100 μL) was added. Absorption at 570 nm was detected on a SpectraMax M5 (Molecular Devices) plate reader. This experiment was performed twice; ED_50_ values are presented as the mean value of these two replicates.

### Clonogenic survival

BxPC3 cells (1,000-3,000) were plated in 6-well plates in growth media and incubated for 4 h at 37°C in 5% CO_2_. Cells were then treated with [^177^Lu]Lu-DOTA-5B1 (0.22 to 1.51 MBq/mL), [^225^Ac]Ac-DOTA-5B1 (0.08 to 5.01 kBq/mL), or sham treated (cell media + radiopharmaceutical vehicle). After 48 h, the treatment-containing supernatant media was removed and cells were washed with PBS. Cells were then incubated with 2.5 mL of growth media for 12 days at 37°C in 5% CO_2_. After 12 days, the media was removed and cells were gently washed with PBS. Cells were then fixed and stained with 3 mL of a mixture of 6% glutaraldehyde and 0.5% crystal violet. Colonies of at least 50 cells (~ 0.5 mm) were counted manually. The plating efficiency, defined as the ratio of the number of colonies formed by the number of cells seeded, was determined using the sham treated cells. The surviving fraction is defined as the number of colonies formed after treatment divided by the number of cells seeded multiplied by the platting efficiency.

### CA19.9 pretargeted radioimmunotherapy

Tumor volumes were monitored with a Peira TM900 imaging device (Peira, Belgium). Four weeks after PDAC cells implantation, mice were randomized into cohorts (n = 8) according to their tumor volume. The mean tumor volume of each cohort was approximately equal to 150 mm^3^. The study was then blinded for the technical person responsible of tumor measurements so that the volume acquired were not biased. One day after randomization, mice receiving PRIT were injected with 5B1-TCO (200 μg, 1 nmol; in 200 μL of PBS). Seventy-two hours later, mice were injected with the appropriate radioactive payload: either [^177^Lu]Lu-DOTA-PEG_7_-Tz (18.5 MBq, 0.4 nmol) or [^225^Ac]Ac-DOTA-PEG_7_-Tz (37 kBq, 0.4 nmol) for PRIT. The control cohorts were injected with either vehicle (0.9 % sterile saline) or 5B1-TCO alone). Tumor volume and body weight were measured twice a week until 120 days or until mice reached the set volume endpoint (> 2000 mm^3^). Mice were monitored for outward signs of toxicity, including lethargy, loss of appetite, or disseminated intravascular coagulation.

### In vivo γH2AX PET imaging

Four weeks post-subcutaneous PDAC cells implantation, mice with an average tumor volume of about 120 mm^3^ were randomized into cohorts (n=5/cohort) and used for PET/CT imaging studies. Three days post-administration of ^177^Lu-/^225^Ac-PRIT (18.5 MBq/0.037 MBq, 0.4 nmol), [^89^Zr]Zr-DFO-anti-γH2AX-TAT (0.5 MBq, 5.0 μg) was administered intravenously. As a positive control, γH2AX was induced in mice by X-ray irradiation of the tumor (X-RAD 320). One hour before the injection of the PET imaging probe, positive control mice were irradiated (10 Gy) at a dose rate of 1.2 Gy/min. As negative control, mice were mock-treated (0 Gy). PET/CT imaging was performed on an Inveon PET/CT scanner (Siemens) up to 4 days post-injection of [^89^Zr]Zr-DFO-anti-γH2AX-TAT. Images were processed using the Inveon Research Workplace software package. Volume of interests (VOIs) analysis was performed using 3D Slicer. VOIs were drawn around the major organs of interest and the tumor. CT scans were used for volume measurements (cm^3^) when PET scans were used for quantification of the activity in the organ of interest. VOIs are presented as percent injected activity per cm^3^ (%IA/cm^3^). After imaging, mice were euthanized; organs were collected, weighted and counted on an automatic gamma counter. Results are presented as the injected activity per gram (%IA/g).

### Ex vivo γH2AX staining

Separate mice cohorts (n=3/cohort) were treated as previously described. However, three days post-injection of the radionuclide therapy, mice were euthanized and their tumors were collected for γH2AX staining. Immunohistochemistry and immunofluorescence were carried out on formalin-fixed, paraffin-embedded 10-μm-thick tumor sections. Sections were submitted to MSKCC Molecular Cytology Core Facility for γH2AX as well as hematoxylin and eosin staining. Slides were scanned using a Pannoramic Flash 250 slide scanner from 3DHistech (Budapest, Hungary) - Zeiss 20x/0.8NA objective. Immunohistochemistry was used for the determination of γH2AX positive cells when immunofluorescence was used for the detection of γH2AX foci per cell. Immunohistochemistry cell positive analysis was performed in random tumor areas (n = 8/sample) using the open source software QuPath 32. The total number of cells was determined using the hematoxylin staining whereas the DAB staining was used to quantify the percentage of positive cells. Immunofluorescence analysis was performed using the open source image processing package Fiji 33. The mean FITC fluorescence intensity was normalized to the DAPI fluorescent signal and the number of foci/cell were counted in random tumor areas (n =6/sample).

### Statistical analyses

All data are represented as mean value ± standard deviation (n = 5-8, unless otherwise noted). The sample sizes were selected taking into account both statistical consideration and the exigencies of funding. For the PET imaging and biodistribution studies, a minimum of 4 animals were required to obtain a statistical power > 0.8 (bilateral test, δ = 1.3, σ = 0.6, α = 0.05). For the therapy studies, a minimum of 8 animals were required to obtain a statistical power > 0.8 (bilateral test, δ = 25, σ = 17, α = 0.05). The significance analyses were performed using Prism 8.3, employing one-way ANOVA followed by a Dunnett's multiple comparisons test and log rank Mantel-Cox test as detailed in figure legends. A P value of <0.05 was considered significant.

## Results

### Exposure to α- and β-RIT results in differential viability in BxPC3 PDAC cell line

**Figure 1:**
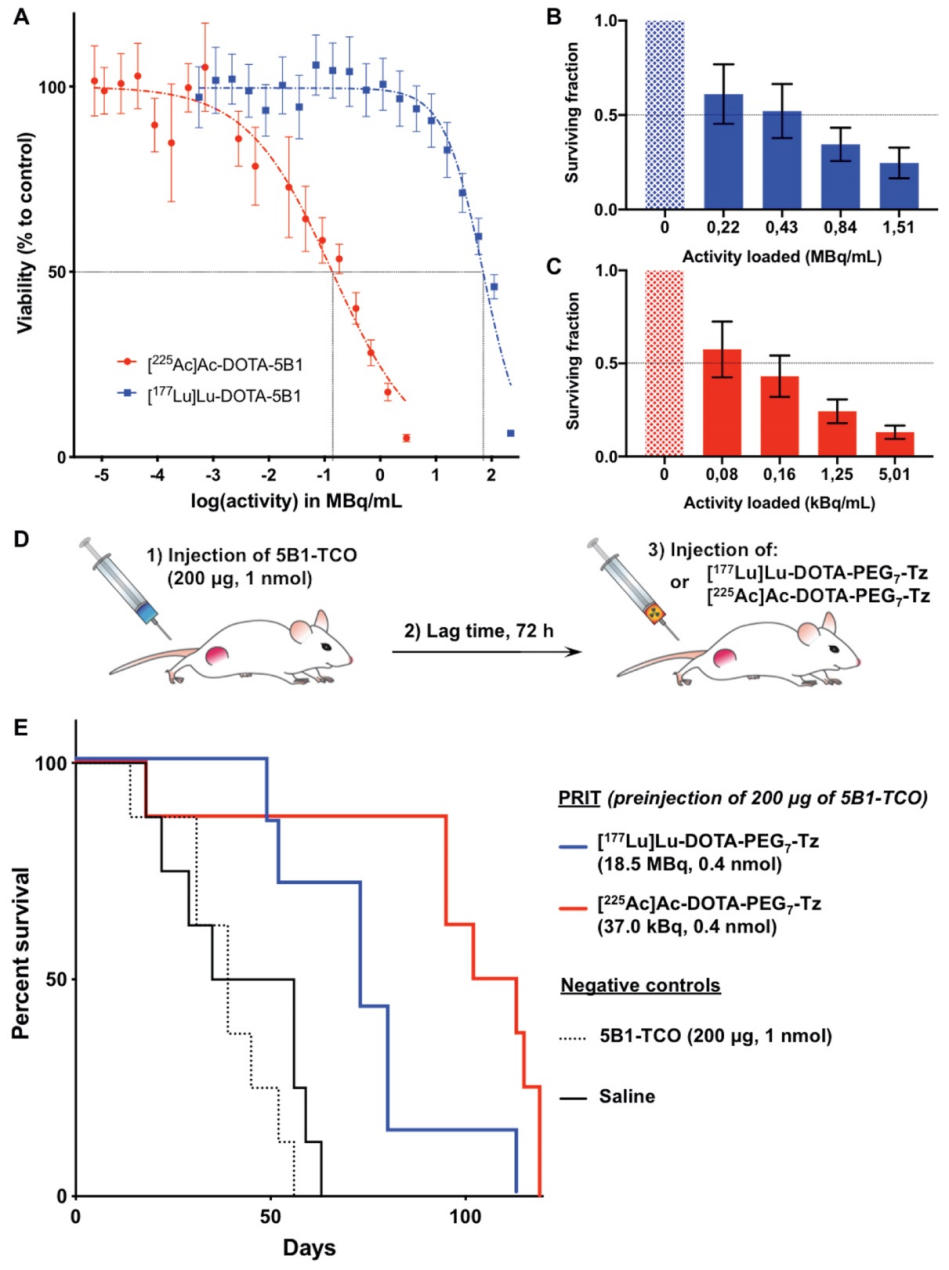
*In vitro* and* in vivo* evidence of the higher cytotoxic potential of α-RIT compared to β-RIT **A.** Viability of BxPC3 PDAC cells after a 48 hours incubation with various activity concentration of [^177^Lu]Lu-DOTA-5B1 and [^225^Ac]Ac-DOTA-5B1 (n=4 per concentration). **B.** Clonogenic survival of BxPC3 cells exposed to [^177^Lu]Lu-DOTA-5B1 (n=6 per concentration). **C.** Clonogenic survival of BxPC3 cells exposed to [^225^Ac]Ac-DOTA-5B1 (n=6 per concentration). **D.** Schematic representation of the PRIT dosing schedule. **E.** Percent survival as function of time after PRIT injection. Mice were sacrificed when tumor >2000 mm^3^. Survival data reflect the progression of primary subcutaneous tumors (n=8 per cohort). Values are represented as means, and error bars represent standard deviations.

Viability of the BxPC3 PDAC cell line after treatment with [^225^Ac]Ac-DOTA-5B1 and [^177^Lu]Lu-DOTA-5B1 was evaluated through an MTT assay. Reduced viability of the BxPC3 cell line was observed after a 48-hour incubation with [^225^Ac]Ac-DOTA-5B1 at activity concentrations greater than 0.002 MBq/mL (*Figure 1A*). The mean ED_50_ value of two replicate experiments was calculated to 0.26 ± 0.17 MBq/mL. After a 48-hour incubation with [^177^Lu]Lu-DOTA-5B1, a reduced cell viability was observed as well. However, much higher activity concentrations (> 8 MBq/mL) were required (*Figure 1A, Figure S1*). The ED_50_ value after [^177^Lu]Lu-DOTA-5B1 treatment was calculated to 122 ± 74 MBq/mL. The ratio of cytotoxic effects between the α- and β-RIT treatments was calculated to be 483 ± 39.

As the method of choice to determine cell reproductive death after ionizing radiation, clonogenic assays were performed and confirmed the higher potency of [^225^Ac]Ac-DOTA-5B1 as compared to [^177^Lu]Lu-DOTA-5B1 (*Figure 1B-C*). Only 0.2 kBq/mL of [^225^Ac]Ac-DOTA-5B1 were required to achieve a surviving fraction of 0.5 (SF_0.5_), when 560 kBq/mL were needed to result in similar effects with [^177^Lu]Lu-DOTA-5B1. The ratio of cytotoxic effects between the α- and β-RIT treatments was here calculated to 2,800.

Based on these two cytotoxic experiments and the different physical properties of the radionuclides used, the relative biological effectiveness between our α- and β-RIT was determined (see SI for calculation details). In the conditions of our MTT assay, [^225^Ac]Ac-DOTA-5B1 was 3.7 ± 0.3 times more cytotoxic than [^177^Lu]Lu-DOTA-5B1. Comparatively, a 21-fold ratio was found between [^225^Ac]Ac-DOTA-5B1 and [^177^Lu]Lu-DOTA-5B1 in the settings of our clonogenic experiment (SF_0.5_). The variability of the relative biological effectiveness between α- and β-RIT calculated for these two experiments highlights the importance of the selected survival endpoint.

### α-PRIT results in prolonged survival as compared to β-PRIT without sign of toxicity

For α- and β-RIT therapy studies, the pretargeting administration method was chosen due to its demonstrated potency and its ability to reduce absorbed dose to healthy tissues and thereof toxicities (*Figure 1D*). Based on the aforementioned *in vitro* assays and our previously published PRIT studies with ^177^Lu and ^225^Ac, the injected activities for [^177^Lu]Lu-DOTA-PEG_7_-Tz and [^225^Ac]Ac-DOTA-PEG_7_-Tz were set to 18.5 MBq and 0.037 MBq, respectively 5,6. With these injected activities, a factor of 500, in the same range as the ratio observed for equitoxic effects in our MTT assay, applies between the ^177^Lu- and ^225^Ac-PRIT. For both PRIT approaches, the injected mass of 5B1-TCO and Tz-radioligand were equal. Our group previously reported similar pharmacokinetics and tumoral uptake between the α- and β-PRIT strategies 5,6. The absorbed dose delivered to the tumor was estimated using mouse dosimetry extrapolated from biodistribution studies previously performed with ^177^Lu-/^225^Ac-PRIT 5,6. The tumor absorbed dose was evaluated to 102 Gy for ^177^Lu-PRIT and 75 Gy for ^225^Ac-PRIT. The accepted threshold for a meaningful response with EBRT in solid tumors being 100 Gy, a significant therapeutic effect was expected for both PRIT treatment group. Control groups were injected with either saline (vehicle) or 5B1-TCO alone.

Survival analysis suggested therapeutic effects in both PRIT cohorts compared with control groups (P values <0.01) (*Figure 1E*). Median survival for the group treated with ^177^Lu-PRIT (73.0 days) and ^225^Ac-PRIT (107.5 days) highlighted the superior therapeutic potential of α-PRIT in our setting (P value < 0.05). Mean tumor volume are provided in supporting information and correlated with the survival results (*Figure S2*). Even though, the tumor absorbed dose was 25 % less in the α-PRIT cohort, the higher therapeutic efficacy of this treatment as compared to β-PRIT suggests that the relative biological effectiveness of 5 recommended by the US Department of Energy for effective dose calculations with α-particle emitters might be under-estimated. The body weight progression for ^225^Ac-PRIT showed a transient deviation from control groups and the ^177^Lu-PRIT cohort from day 3 to day 21 after the administration of the systemic radiotherapy (*Figure* S2). Nevertheless, no weight loss exceeded the 20% threshold that would have required the early euthanasia of the animal. No other sign of gross acute toxicity was observed. All the mice were sacrificed when their tumor reached the set endpoint of > 2000 mm^3^ or at 120 days.

### Exposure to β-PRIT in BxPC3 PDAC xenografts induces increased uptake of [^89^Zr]Zr-DFO-anti-γH2AX-TAT as compared to controls

**Figure 2:**
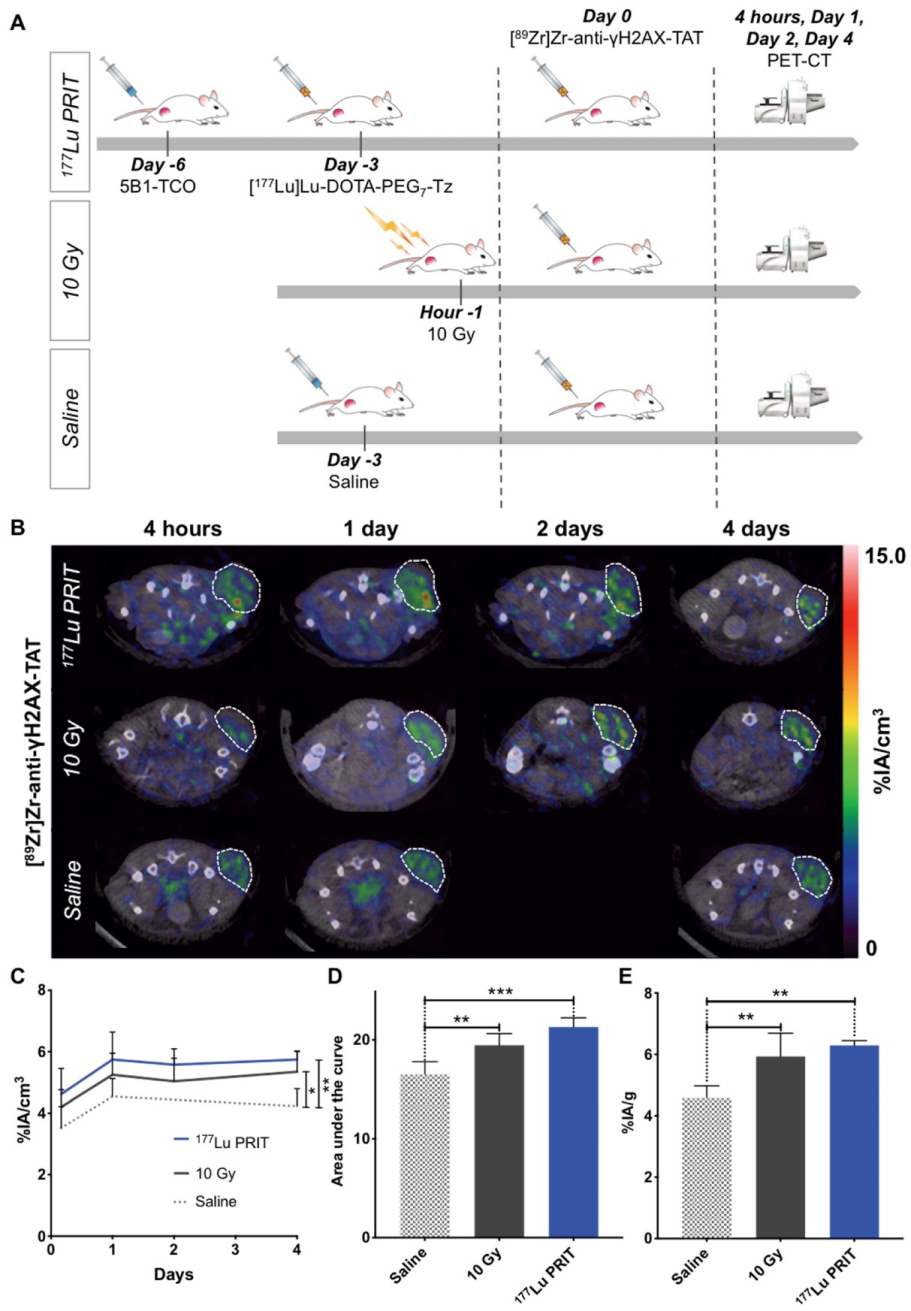
*In vivo* imaging of DNA damage with [^89^Zr]Zr-DFO-anti-γH2AX-TAT following β-PRIT in a PDAC mouse model. **A.** Schematic representation of the β-PRIT and ^89^Zr-PET imaging schedule in subcutaneous BxPC3 xenografted athymic nude mice. **B.** Representative transverse PET/CT images up to 4 days post-intravenous injection of [^89^Zr]Zr-DFO-anti-γH2AX-TAT (n=4 per cohort). Tumors are delineated with white dashed lines. **C.** Tumor VOI analysis of the PET/CT images up to 4 days p.i. (n=4 per cohort). **D.** Area under the curve of the tumor VOI analysis between 4 hours and 4 days p.i. (n=4 per cohort). **E.** Tumor uptake as determined through ex vivo gamma-counting post-animal sacrifice (n=5 per cohort). Values are represented as means, and error bars represent standard deviations. One-Way ANOVA followed by a Dunnett's multiple comparisons test was applied. Adjusted P values: *** P ≤ 0.001, ** P≤ 0.01, * P≤ 0.05, n.s. = non significant.

In an attempt to measure *in vivo* the response to ^177^Lu-PRIT, [^89^Zr]Zr-DFO-anti-γH2AX-TAT was used to visualize DNA damage via PET imaging. Mice were treated with ^177^Lu-PRIT (18.5 MBq) 72 hours prior the injection of the PET anti-γH2AX tracer to allow sufficient dose deposition and induction of γH2AX at the tumor site (*Figure 2A*) 34. Positive and negative control cohorts were treated according to established protocols (*Figure 2A*). PET images were recorded at multiple time points after the injection of the PET tracer to define the best time point for the visualization of DNA damage after PRIT. Representative transverse images are shown in *Figure 2B*, coronal sections and maximum intensity projections are shown in the supporting information (*Figure S3*). VOI analyses of tumors were performed at the different time points (*Figure 2C, Figure S4A*). At the 4 days imaging time point, a statistically significant (P value < 0.01) increased uptake of [^89^Zr]Zr-DFO-anti-γH2AX-TAT was observed in the ^177^Lu-PRIT cohort as compared to the negative control. An increasing uptake trend was also observed with the positive control (10 Gy) cohort (P < 0.05). Analysis of the area under the curve revealed a statistically significant increased accumulation of [^89^Zr]Zr-DFO-anti-γH2AX-TAT over the 4 day imaging period in both 10 Gy and ^177^Lu-PRIT cohorts (*Figure 2D*). In every cohort, the uptake of [^89^Zr]Zr-DFO-anti-γH2AX-TAT in the tumor increases up to 24 hours post-injection of the ^89^Zr-PET radiotracer before reaching a plateau. Consequently, the 24 hours imaging time point will as of now be used in this study for the *in vivo* evaluation of DNA damage with [^89^Zr]Zr-DFO-anti-γH2AX-TAT. After the final image acquisition, mice were sacrificed and organs of interest were counted on a γ-counter to confirm the PET imaging results. The biodistribution confirmed the significantly higher accumulation of [^89^Zr]Zr-DFO-anti-γH2AX-TAT in irradiated tumors (*Figure 2E*). Cohorts irradiated with 10 Gy of external beam radiation or ^177^Lu-PRIT showed increased retention of the ^89^Zr-PET anti-γH2AX tracer with uptake of 5.93 ± 0.76 and 6.29 ± 0.15 %IA/g, respectively. The uptake in our negative control cohort was significantly lower with 4.58 ± 0.76 %IA/g. The full biodistribution profile of [^89^Zr]Zr-DFO-anti-γH2AX-TAT (*Figure S4C*) matches the expectations for a full-length IgG radioimmunoconjugates with an hepatobiliary clearance and prolonged half-life in the blood and vascularized organs. We observed a significantly higher accumulation of [^89^Zr]Zr-DFO-anti-γH2AX-TAT in the blood, well-perfused organs (e.g. heart, lungs) and in the spleen of irradiated animals (*Figure S4B-C*). In the case of the ^177^Lu-PRIT cohort, the uptake of [^177^Lu]Lu-DOTA-PEG_7_-Tz in the blood 72 hours post-injection is 1.51 ± 0.21 %IA/g and the mean absorbed dose was calculated to be 48.8 cGy/MBq, therefore approximately 9.0 Gy was delivered to the blood 5. Even though no hematotoxicity was reported in our previous studies with ^177^Lu-PRIT, such a dose could result in the generation of off-target toxicities and therefore elevated γH2AX level in the blood. In the case of the 10 Gy external beam radiation cohort, the mice were positioned on the outside X-ray field and only their lower right side were exposed to the radiation. Therefore, according to the dose delivered and their position we didn't expected any significant toxicity to the blood. The radiation exposure is consequently not expected to be the source of the increased uptake of ^89^Zr-PET anti-γH2AX tracer.

### α-PRIT results in an increased [^89^Zr]Zr-DFO-anti-γH2AX-TAT tumoral uptake that correlates with the prolonged survival of the animals as compared to β-PRIT

**Figure 3:**
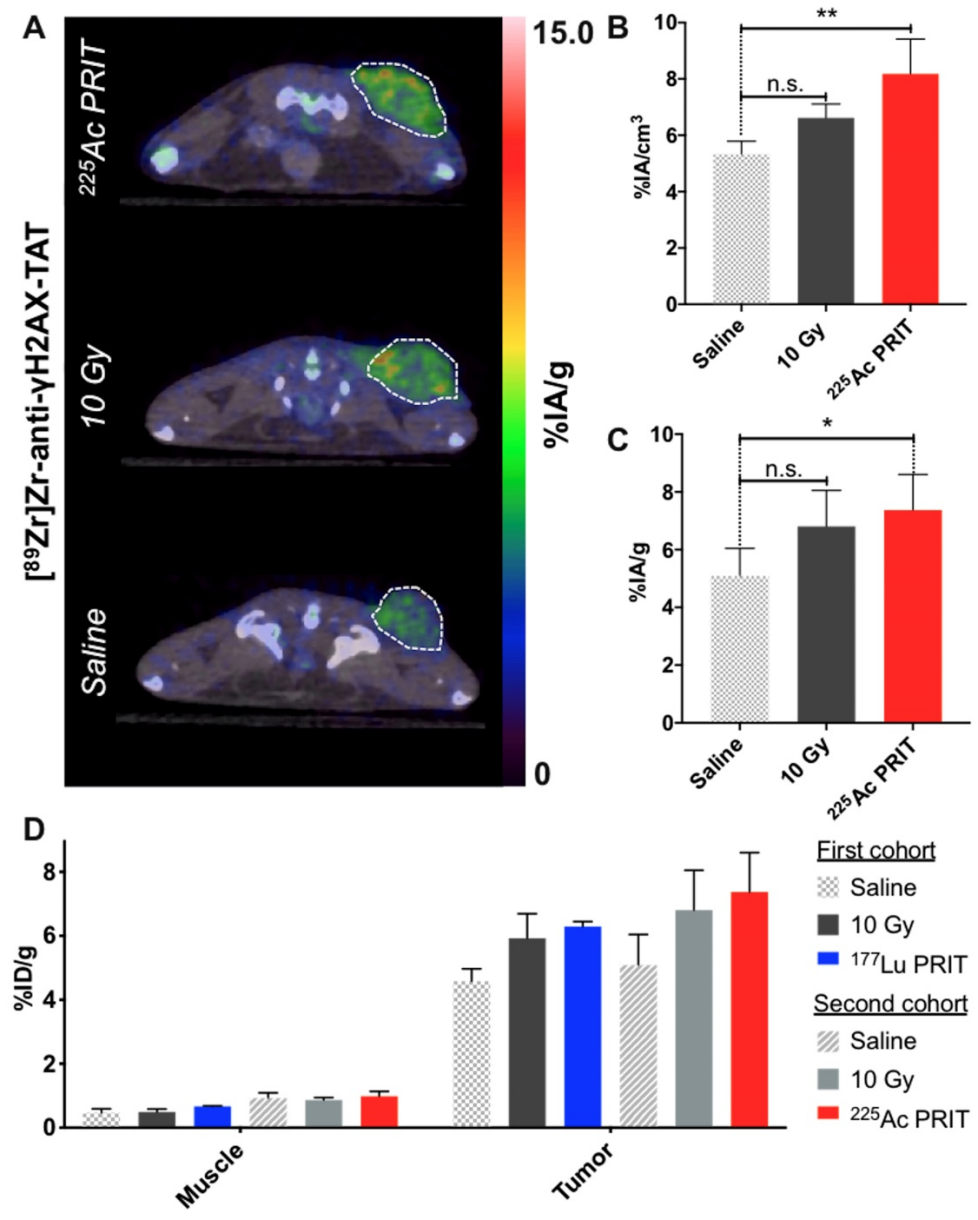
*In vivo* imaging of DNA damage with [^89^Zr]Zr-DFO-anti-γH2AX-TAT following α-PRIT in a PDAC mouse model. Comparison with β-PRIT **A.** Representative transverse PET/CT images 24 hours post-intravenous injection of [^89^Zr]Zr-DFO-anti-γH2AX-TAT (n=4 per cohort). Tumors are delineated with white dashed lines. **B.** Tumor VOI analysis of the PET/CT images 24 hours p.i. (n=4 per cohort). **C.** Tumor uptake as determined through ex vivo gamma-counting post-animal sacrifice (n=5 per cohort). **D.** Comparison of the β-PRIT and α-PRIT DNA damage imaging cohorts in terms of [^89^Zr]Zr-DFO-anti-γH2AX-TAT tumor and muscle uptake as determined through ex vivo gamma-counting post-animal sacrifice (4 days and 24 hours post-administration of the PET radiotracer, respectively). Values are represented as means, and error bars represent standard deviations. One-Way ANOVA followed by a Dunnett's multiple comparisons test was applied. Adjusted P values: ** P≤ 0.01, * P≤ 0.05, n.s. = non significant.

Following the protocol established with ^177^Lu-PRIT, PET imaging of DNA damage following ^225^Ac-PRIT (37 kBq) was undertaken. Representative transverse images of [^89^Zr]Zr-DFO-anti-γH2AX-TAT uptake at the tumor site 24 hours post-intravenous injection of the PET radiotracer are reported in *Figure 3A*. Coronal sections and maximum intensity projections are provided in supporting information (*Figure S5A*). Quantitative VOI analysis of the ^89^Zr-PET signal revealed a statistically significant (P < 0.05) increase of the tumor [^89^Zr]Zr-DFO-anti-γH2AX-TAT uptake in the ^225^Ac-PRIT cohort (*Figure 3B*). VOI analysis was compared to the results determined through ex vivo gamma-counting post-animal sacrifice (*Figure S6*). The uptake of the ^89^Zr-PET DNA damage radiotracer in the ^225^Ac-PRIT and positive control (10 Gy) cohorts was 7.37 ± 1.23 and 6.80 ± 1.24 %IA/g, respectively, when the negative control cohort uptake was 5.08 ± 0.95 %IA/g (*Figure 3C, Figure S5B*). These results were in perfect agreement with the PET VOI values.

Comparing the ^225^Ac-PRIT and ^177^Lu-PRIT imaging cohorts is rendered difficult due to confounding factors that include different animal cohorts, PET radiotracer synthesis, or PET imaging endpoint (4 days post-radiotracer administration for ^177^Lu-PRIT and 24 hours for ^225^Ac-PRIT). However, there is no evidence of difference between the two different negative and positive control cohorts. No statistically significant differences can be observed between the ^225^Ac-PRIT and ^177^Lu-PRIT cohorts with our DNA damage PET imaging tracer (*Figure 3D*), even though ^225^Ac-PRIT resulted in prolonged survival of the treated animal as compared to ^177^Lu-PRIT. This study seems to indicate that DNA damage is not the only apoptotic pathway involved in the therapeutic efficacy of our PRIT approaches.

### Ex vivo γH2AX analysis correlates with in vivo ^89^Zr-anti-γH2AX PET/CT imaging

**Figure 4:**
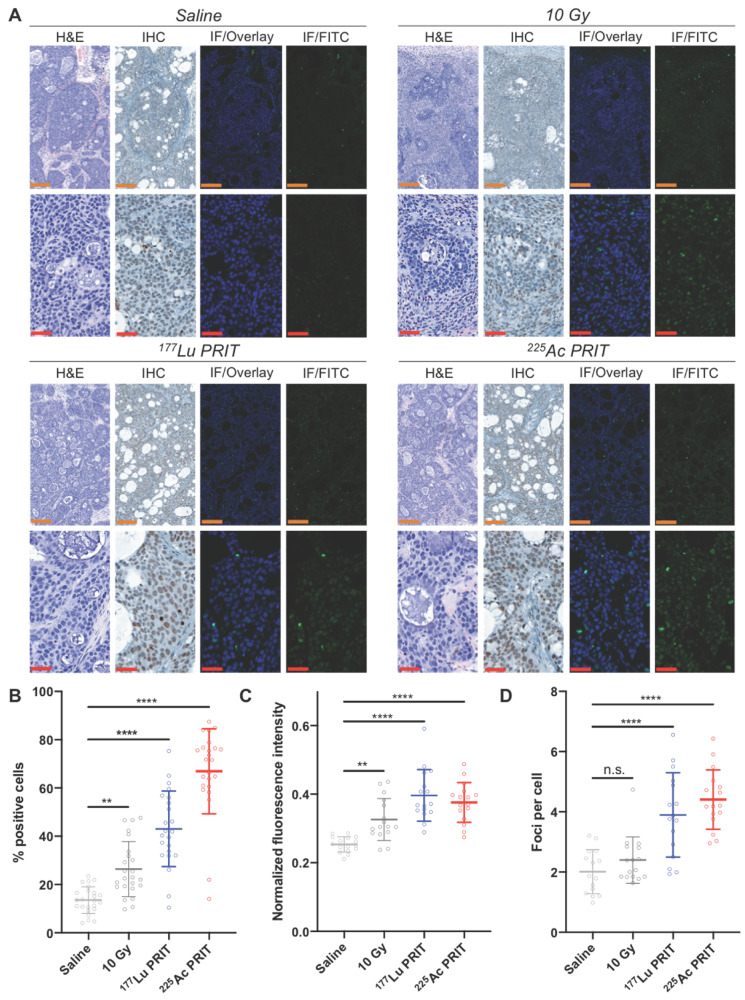
Ex vivo γH2AX immunostaining for the monitoring of α-/β-PRIT in tumor sections of PDAC mouse model 72 hours post-irradiation (n=3 per cohort). **A.** Representative microscopy images (orange scale = 200 μm, red scale = 50 μm). Zoomed-in area are provided in Supporting information (*Figure S8*) **B.** Percentage of γH2AX positive cells after immunohistochemistry analysis of tumor sections following α-/β-PRIT as compared to negative and positive controls (n=8 per slide). Quantification of: **C.** γH2AX fluorescent signal normalized to DAPI signal (n=8 per slide), and **D.** γH2AX foci per cell (n=6 per slide), after immunofluorescence analysis of tumor sections following α-/β-PRIT as compared to negative and positive controls. Values are represented as means, and error bars represent standard deviations. One-Way ANOVA followed by a Dunnett's multiple comparisons test was applied. Adjusted P values: **** P≤ 0.0001, ** P≤ 0.01, n.s. = non significant.

PET/CT results were confirmed via ex vivo immunohistochemistry and fluorescence staining of γH2AX foci in tumor sections following treatment with α-PRIT (37 kBq) and β-PRIT (18.5 MBq) (*Figure 4A, Figure S7*). With immunohistochemistry, the percentage of cells staining positive for γH2AX expression was significantly higher (P < 0.0001) in the cohort treated with α- and β-PRIT as well as in the 10 Gy positive control cohort (P < 0.01) when compared to the saline negative control cohort (*Figure 4B*). ^225^Ac-PRIT treated tumors showed the highest percentage of γH2AX positive cells (66.9 ± 17.6 %) followed by the ^177^Lu-PRIT treated tumors (43.1 ± 15.7 %), the 10 Gy cohort (26.4 ± 11.4 %), and finally the negative control (13.5 ± 5.51 %). Similarly, immunofluorescence γH2AX staining revealed increased fluorescence intensity and number of γH2AX foci per cell in the α-/β-PRIT treated cohorts (P < 0.0001). Notably, the detected number of γH2AX foci was twice as high in α-/β-PRIT cohorts (4.7 ± 1.0 and 4.2 ± 1.4 foci/cell, respectively) as compared to the saline treated cohort (1.9 ± 0.9 foci/cell).

## Discussion

There is presently no established non-invasive clinical approach for the local and systemic evaluation of radiation-induced biomarkers following targeted radionuclide therapy. Instead, targeted radionuclide therapies are not personalized to individual radiation response but rather administered at a set activity/kg based on observed general population response. When the field of targeted radionuclide therapy should transition to a personalized approach based on dosimetry, it is critical to develop in parallel non-invasive approaches for the evaluation of radiation-induced biomarker for early assessment and prediction of therapeutic response. Herein, we have demonstrated that ^89^Zr-PET imaging of DNA damage response is an effective non-invasive tool for the monitoring of the early therapeutic response following α- and β-particle molecular radiotherapy in a preclinical mouse model of PDAC.

First, we validated that exposure to CA19.9 targeted α- and β-particle molecular radiotherapy resulted in reduced viability and clonogenic survival in BxPC3 PDAC cells. Using the pretargeting administration method 5,6, we confirmed *in vivo* the higher therapeutic potential of the CA19.9 pretargeted α-particle radiotherapy as compared to β-particle radiotherapy, when administered in activity ratios that resulted in equitoxic effects *in vitro*. Such results are not surprising due to the higher RBE of α-particle and correlates with results previously reported with ^225^Ac- and ^177^Lu-somatostatin receptor targeted radiotherapy 35. However, they highlight the need for an *in vivo* predictor of radiation-induced effects, independent of the radiation type.

PET imaging studies performed following β-particle PRIT in a BxPC3 PDAC subcutaneous xenograft mouse model allowed the establishment of an imaging protocol that enabled the monitoring of the tumor radiobiological response through γH2AX measurement. This non-invasive molecular imaging approach was successfully translated to α-particle PRIT. This study further highlights the versatile potential of the [^89^Zr]Zr-DFO-anti-γH2AX-TAT radiotracer for the imaging of response following the induction of DNA double-strand break damage from multiple sources (EBRT, α/β-particle) 25,29. No evidence of a difference in [^89^Zr]Zr-DFO-anti-γH2AX-TAT uptake at the tumor site after α-PRIT and β-PRIT could be observed when α-PRIT resulted in prolonged survival compared to β-PRIT. This lack of evidence could be due to inter-experiment confounding factors but more importantly highlights that DNA damage is not the sole radiobiology paradigm and that off-targeted (bystander) effects should be considered when trying to compare different targeted radionuclide therapy approaches 36. Our *in vivo* results were confirmed later with ex vivo γH2AX immunohistochemistry and immunofluorescence staining. Non-invasive therapeutic response assessment is critical at the preclinical stage to validate the potential of novel molecular radiotherapy approaches and expedite the clinical translation phase. In addition, such approach offers great clinical prospects as an *in vivo* biodosimeter for treatment response assessment at the molecular level to better tailor therapeutic regimens to patient response and elucidate the heterogeneous response observed between patients but also between different tumor sites or within the same tumor. The first clinical evaluation of this DNA damage radiotracer should investigate the correlation of early [^89^Zr]Zr-DFO-anti-γH2AX-TAT PET signal following targeted radionuclide therapy or EBRT to patient response evaluated at later time point according to the conventional RECIST/PERCIST criteria. Such evaluation should allow us to determine a γH2AX PET signal cut-off between responders and non-responders.

A major limitation for the clinical translation and use of molecular radiotherapy approaches lies in the irradiation of healthy tissues following target engagement (on-target toxicity) or the non-specific accumulation of the radiopharmaceutical (off-target toxicity) 37,38. γH2AX stands as a potential biomarker for radiation-induced toxicities and allowed previously the detection of radiotherapy-induced DNA damage in healthy human tissues, such as peripheral blood lymphocytes, hair follicles or kidneys 39-41. Our DNA damage PET imaging should therefore allow the monitoring of on-/off-target toxicities. Our study did not allow such observation. Increased accumulation of our PET tracer in the blood and vascularized tissues was observed in the ^177^Lu-PRIT and first EBRT cohort, however this phenomenon was unlikely radiation-induced as it was not observed with the second EBRT cohort or with ^225^Ac-PRIT. Further preclinical studies should evaluate the potential of DNA damage PET imaging after targeted radionuclides therapies that demonstrate known toxicities to healthy tissues.

In conclusion, we have demonstrated in a preclinical PDAC mouse model the potential of [^89^Zr]Zr-anti-γH2AX-TAT as an *in vivo* biodosimeter for α- and β-particle targeted radionuclide therapy response assessment.

## Supplementary Material

Supplementary methods and figures.
