# Supplementary material for: 89Zr-PET imaging of DNA double-strand breaks for the early monitoring of response following α- and β-particle radioimmunotherapy in a mouse model of pancreatic ductal adenocarcinoma
Source: Theranostics. 2020 Apr 27;10(13):5802–14. doi: 10.7150/thno.44772 (PMC7255009; doi:10.7150/thno.44772)
Supplement: Supplementary file 1 — Supplementary methods and figures. [file thnov10p5802s1.pdf]

Supporting information - ^{89}Zr-PET imaging of double DNA strand breaks for the early monitoring of response following α- and β-particle radioimmunotherapy in a mouse model of pancreatic ductal adenocarcinoma

Sophie Poty[1]*, Komal Mandeylwala[1], Edward O'Neill[2], James Knight[2], Bart Cornelissen[2], Jason S. Lewis[1]*.

[1] Department of Radiology, Memorial Sloan Kettering Cancer Center, NY, USA

[2] CRUK/MRC Oxford Institute of Radiation Oncology, Department of Oncology, University of Oxford, Oxford, UK

[3] Radiochemistry and Molecular Imaging Probes Core, Memorial Sloan Kettering Cancer Center, New York, NY, USA

[4] Department of Pharmacology, Weill Cornell Medical College, New York, NY, USA

[5] Molecular Pharmacology Program, Memorial Sloan Kettering Cancer Center, New York, NY, USA

[6] Department of Radiology, Weill Cornell Medical College, New York, NY, USA

Methods

Relative biological effectiveness calculation

The relative biological effectiveness (RBE) was calculated based on 48 hours in vitro cytotoxicity experiments as followed:

$$RBE = \frac{Ratio\ of\ cytotoxicity}{Ratio\ of\ total\ particle\ energy\ per\ decay\ at\ 48\ hours}$$

The ratio of total particle energy per day is calculated taking into account the different physical properties of lutetium-177 and actinium-225 as followed:

- Lutetium-177 ($t_{1/2}$ = 6.7 days, total energy per decay = 0.147 MeV)
- Actinium-225 ($t_{1/2}$ = 9.9 days, total energy per decay [including decay daughters] = 28.16 MeV)

$$Ratio\ (48\ hours) = \frac{{}^{225}Ac\ total\ energy\ per\ decay\ (48\ hours)}{{}^{177}Lu\ total\ energy\ per\ decay\ (48\ hours)}$$

$$= \frac{5.688}{0.044} = 129$$

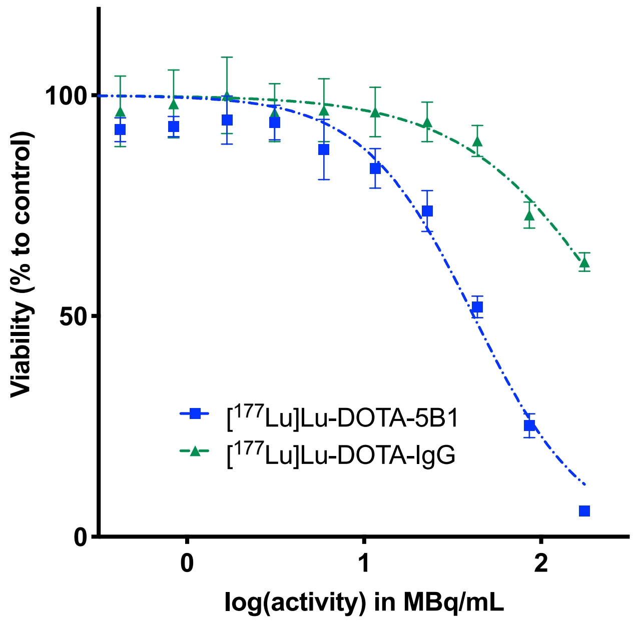

Figure S1. *In vitro* cytotoxic potential of β-RIT**.** Viability of BxPC3 PDAC cells after a 48 hours incubation with various activity concentration of [^{177}Lu]Lu-DOTA-5B1 as compared to [^{177}Lu]Lu-DOTA-IgG (n=3 per concentration). Non-specific effect observed here are partly due to cross-fire effect.

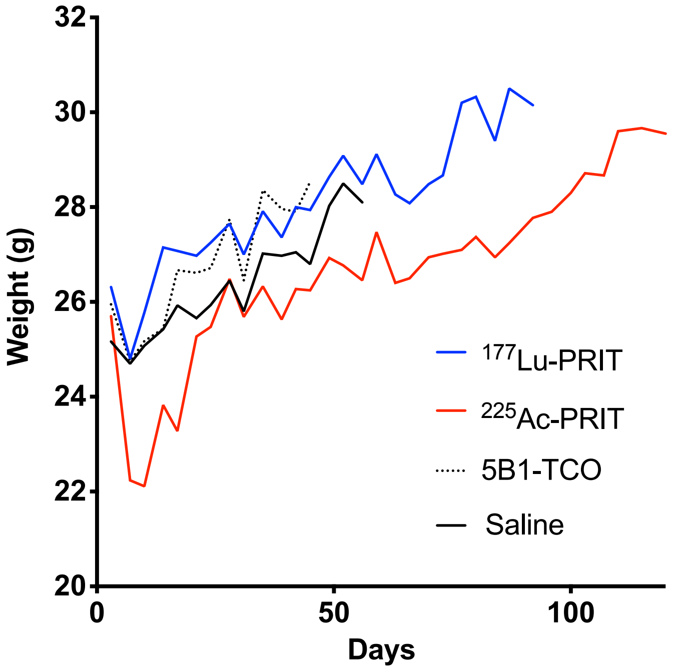

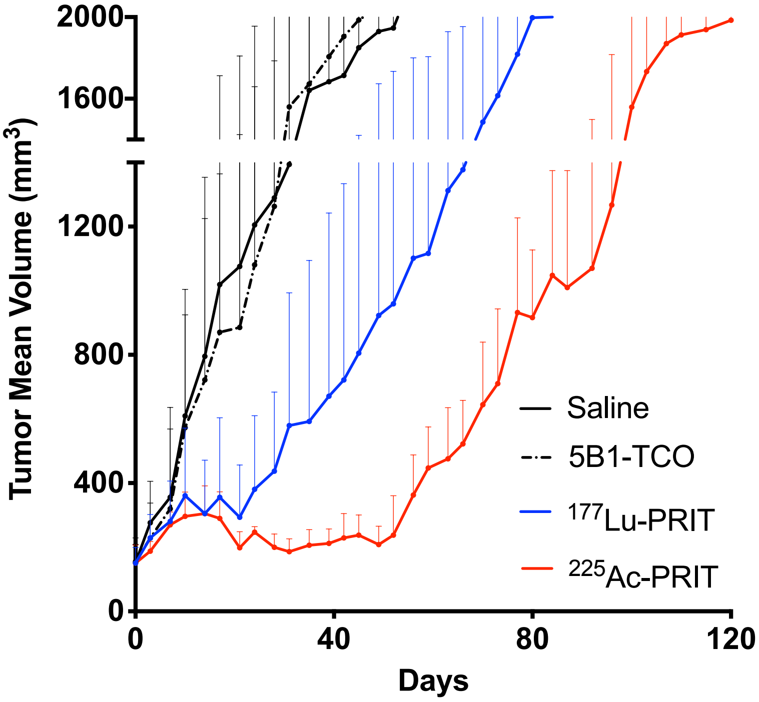

Figure S2. Top. Change in body weight during the course of β- and α-PRIT. Only the mean value at each time point is represented for more clarity. The mean weights values are presented until n = 2 in each cohort. Bottom. Mean tumor volume during the course of β- and α-PRIT.

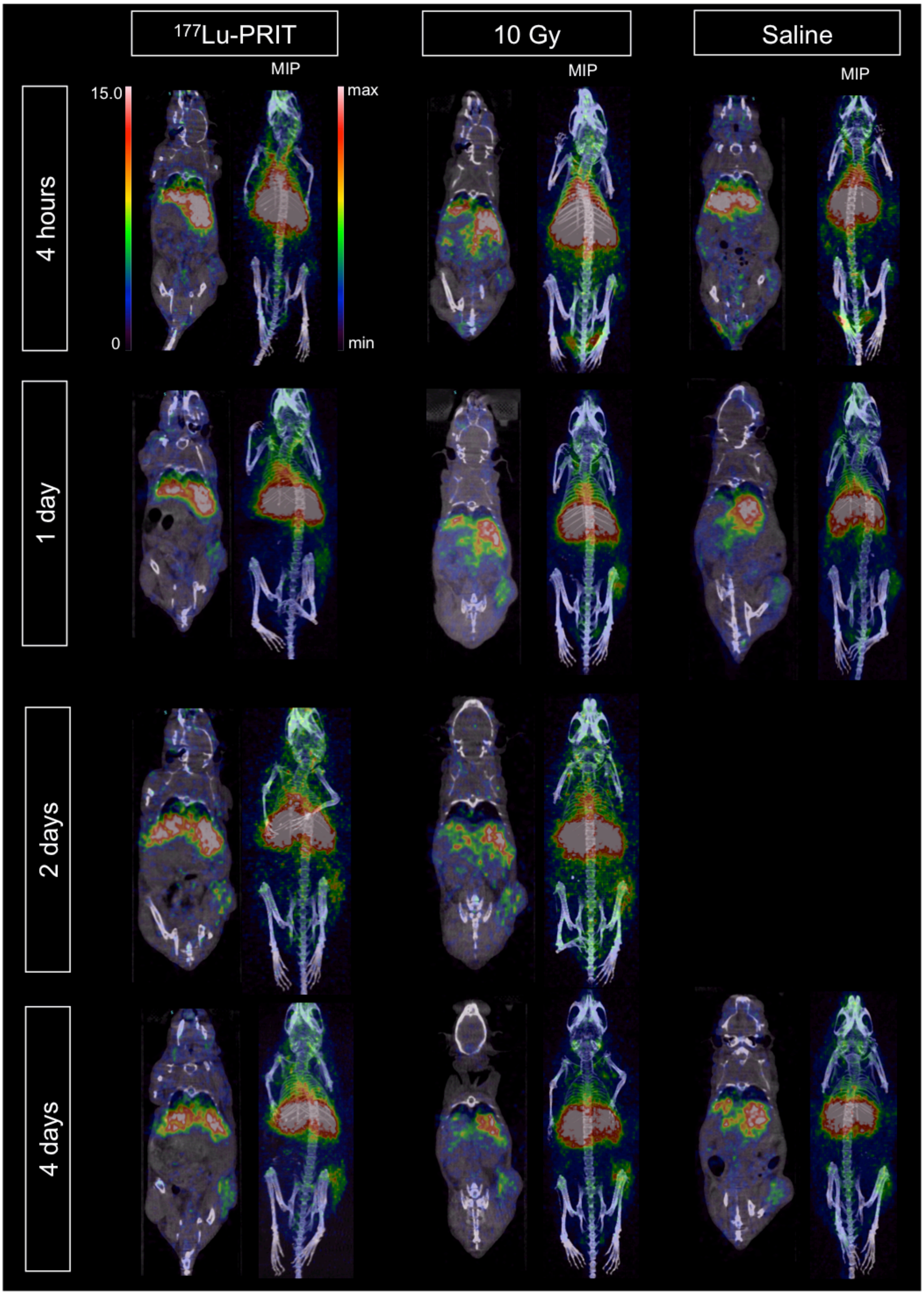

Figure S3. PET/CT images showing coronal sections (left) and maximum intensity projections (right) of [^{89}Zr]Zr-DFO-anti-γH2AX-TAT following β-PRIT in a BxPC3 subcutaneous PDAC xenograft mouse model.

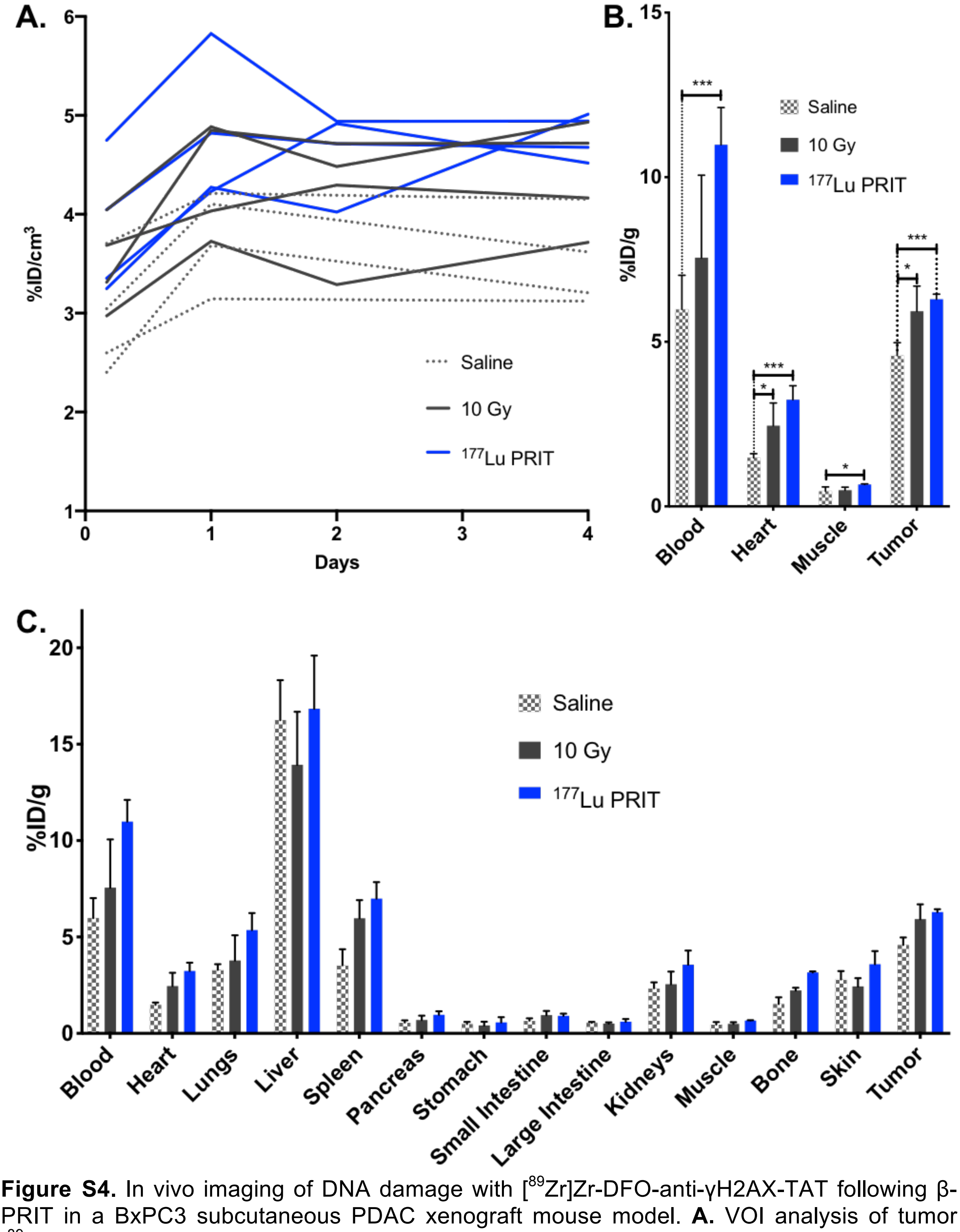

Figure S4. In vivo imaging of DNA damage with [^{89}Zr]Zr-DFO-anti-γH2AX-TAT following β-PRIT in a BxPC3 subcutaneous PDAC xenograft mouse model. **A.** VOI analysis of tumor [^{89}Zr]Zr-DFO-anti-γH2AX-TAT uptake at the different PET/CT imaging time point. **B.** Organ of interest uptake as determined through ex vivo gamma-counting post-animal sacrifice (4 days post-injection of the PET radiotracer). **C.** Full biodistribution profile of [^{89}Zr]Zr-DFO-anti-γH2AX-TAT in the different cohorts, 4 days post-injection of the PET radiotracer. Values are represented as means, and error bars represent standard deviations. *** $P \leq 0.001$, * $P \leq 0.05$, n.s. = non significant.

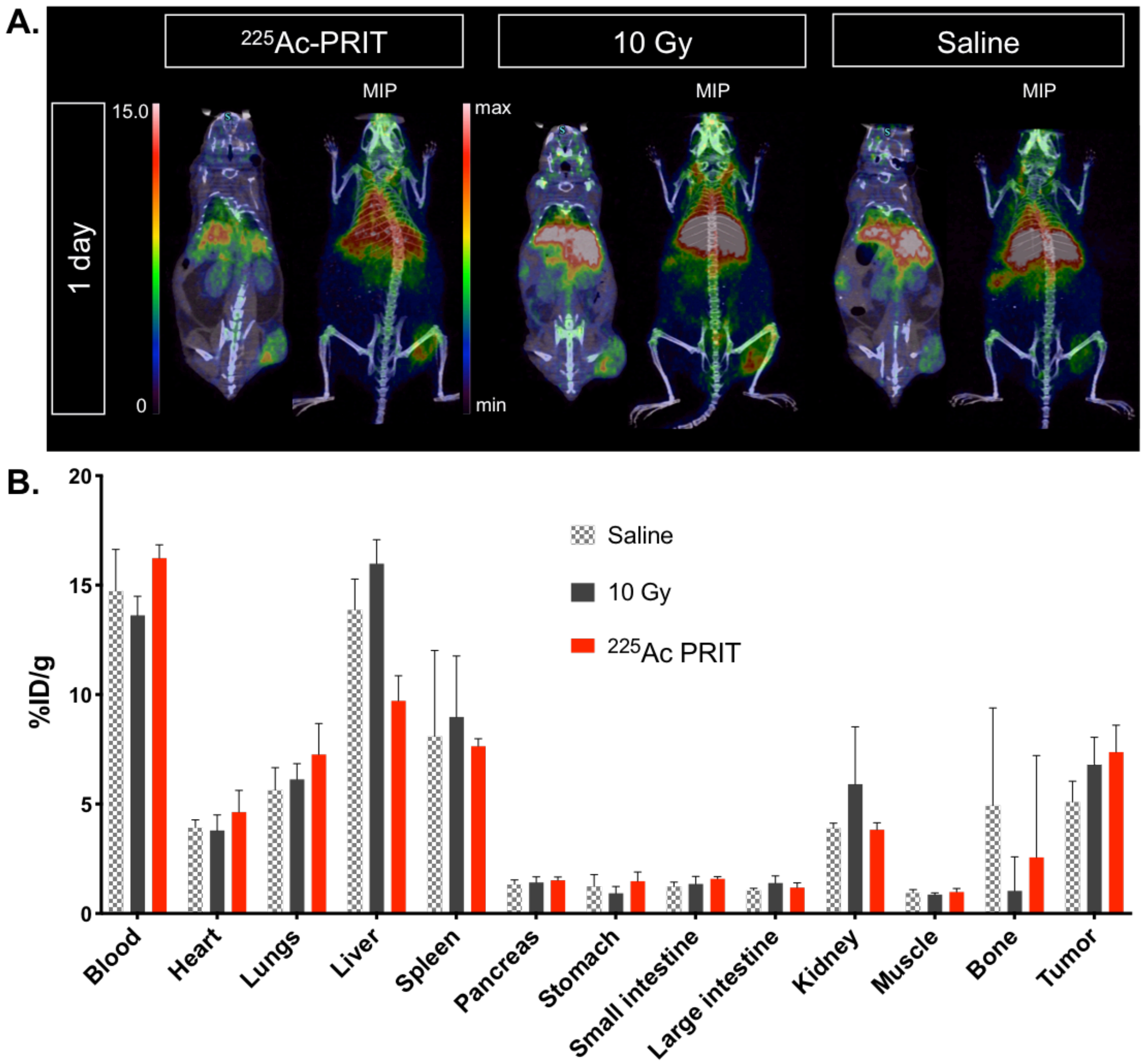

Figure S5. In vivo imaging of DNA damage with [^{89}Zr]Zr-DFO-anti-γH2AX-TAT following α-PRIT in a BxPC3 subcutaneous PDAC xenograft mouse model. **A.** PET/CT images showing coronal sections (left) and maximum intensity projections (right) of [^{89}Zr]Zr-DFO-anti-γH2AX-TAT. **B.** Full biodistribution profile of [^{89}Zr]Zr-DFO-anti-γH2AX-TAT in the different cohorts, 24 hours post-injection of the PET radiotracer. Values are represented as means, and error bars represent standard deviations.

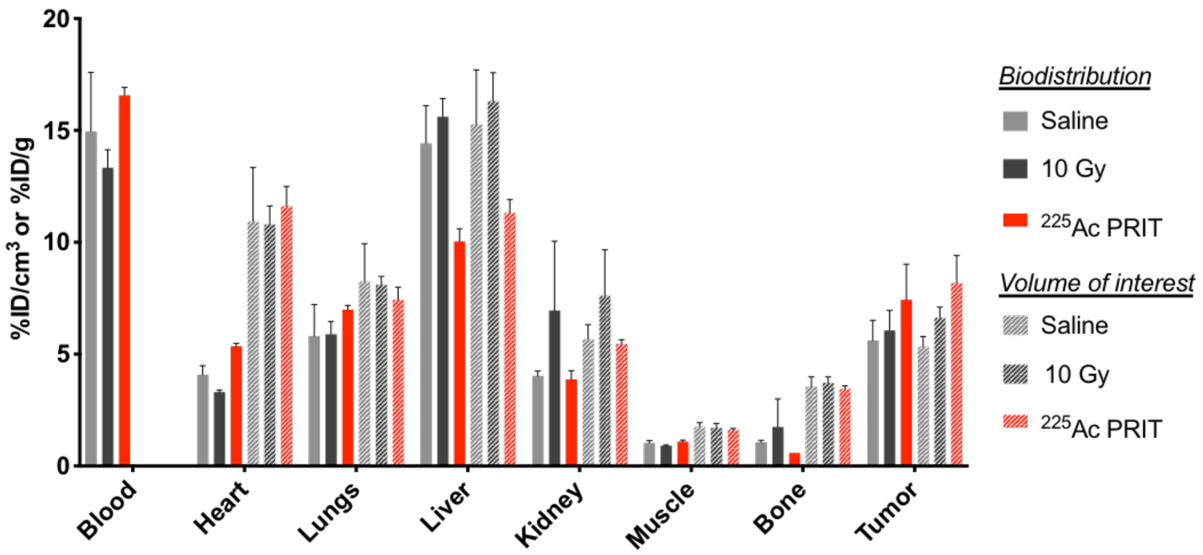

Figure S6. Correlation of VOI analysis and organ uptakes as determined through ex vivo gamma-counting post-animal sacrifice with [^{89}Zr]Zr-DFO-anti-γH2AX-TAT following α-PRIT in a BxPC3 subcutaneous PDAC xenograft mouse model, 24 hours post-injection of the PET radiotracer.

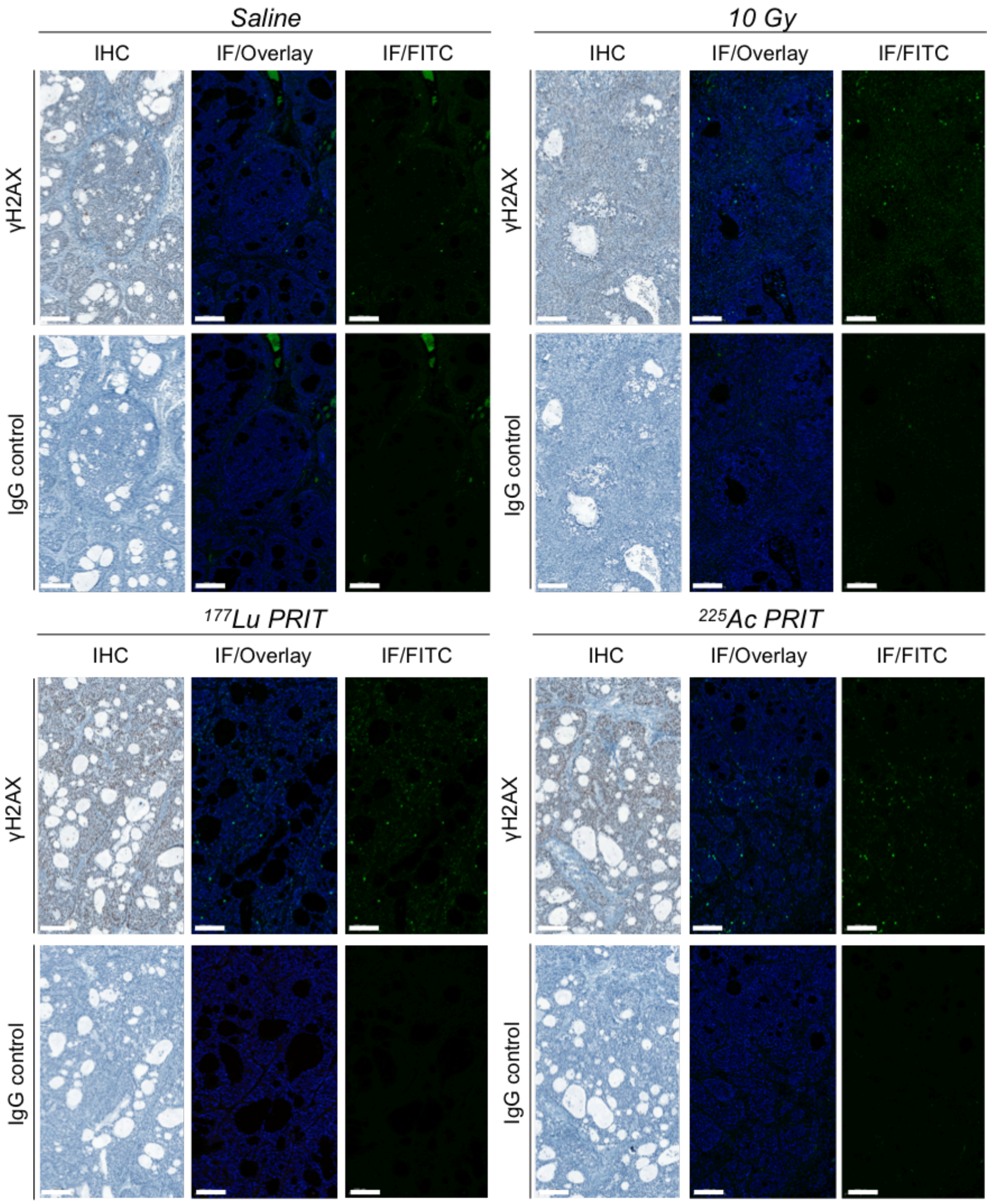

Figure S7. γH2AX immunohistochemistry and immunofluorescence staining of BxPC3 mice subcutaneous xenografts treated with saline (negative control), 10Gy EBRT (positive control), β- or α-PRIT. Top images are representative areas of the staining performed with an anti-γH2AX antibody. Bottom images are representative areas of the staining performed with an IgG isotype control. The comparison highlights the specificity of our staining for γH2AX. Scale bar = 200 µm.

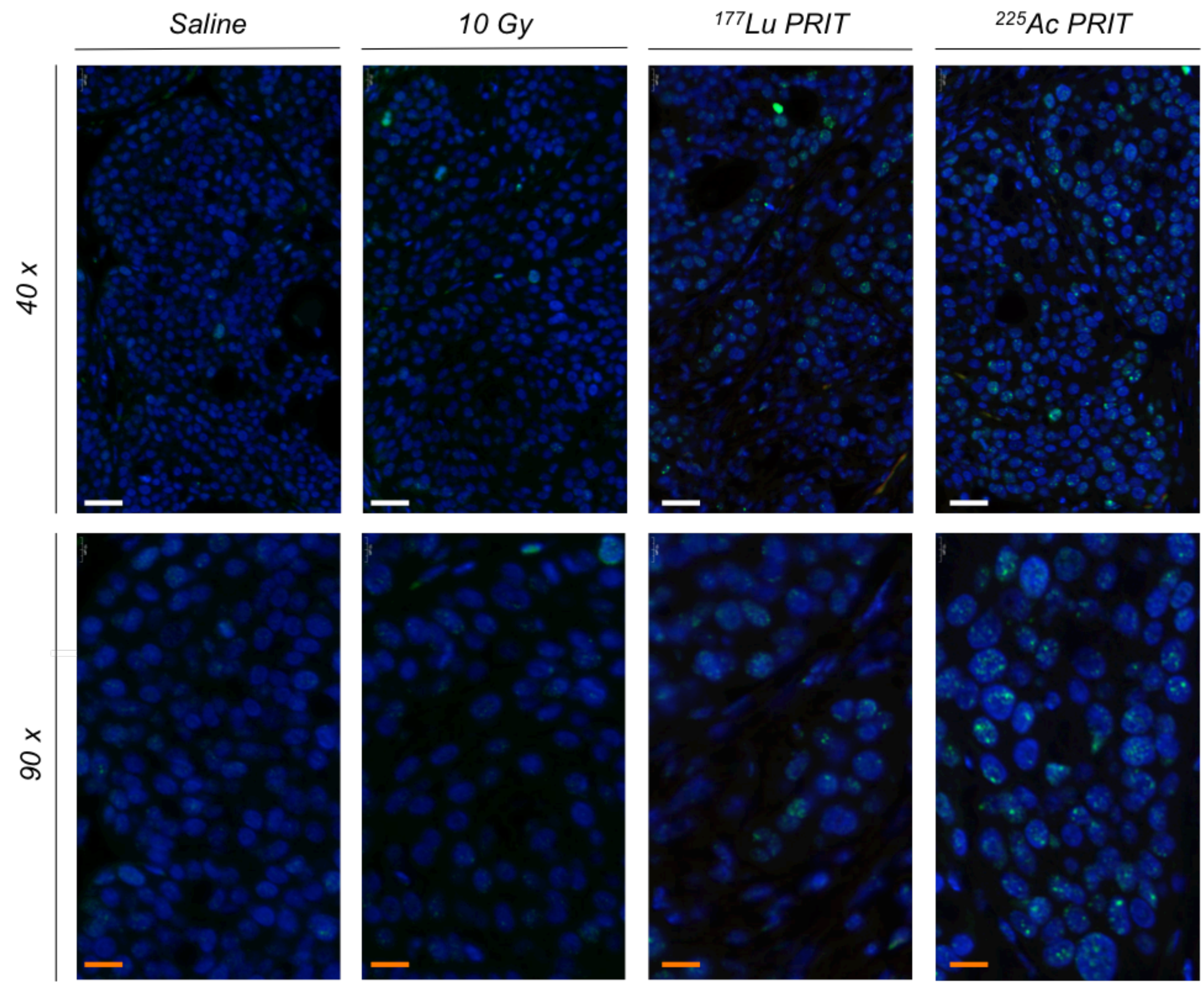

Figure S8. γH2AX immunofluorescence staining of BxPC3 mice subcutaneous xenografts treated with saline (negative control), 10Gy EBRT (positive control), β- or α-PRIT. White scale bar = 40 µm, Orange scale bar = 20 µm.
